# Characterization and phylogenetic analysis of the complete plastome sequence of *Thunia alba* (Lindley) H. G. Reichenbach (Orchidaceae), a rare wild ornamental orchid

**DOI:** 10.1080/23802359.2019.1688729

**Published:** 2019-11-13

**Authors:** Ruiyang Zhang

**Affiliations:** Agriculture & Biological Engineering College, Jinhua Polytechnic, Jinhua, PR China

**Keywords:** *Thunia alba*, plastome, phylogenetic analysis, ornamental orchid

## Abstract

*Thunia alba* (Lindley) H. G. Reichenbach is a wild ornamental orchid, and it is a rare plant species with small populations. In this study, the complete chloroplast genome sequence of *T. alba* was assembled using short reads produced by high-throughput sequencing technologies. The whole chloroplast genome was 159,948 bp in length with a typical quadripartite structure, which consisted of a large single-copy (LSC), a small single-copy (SSC), and two inverted repeats (IRs). The sizes of LSC, SSC, and IR were 87,532, 18,852, and 26781 bp, with GC contents of 35.0%, 30.3%, and 43.2%, respectively. There were a total of 135 genes, which included 88 protein-coding genes, 8 rDNA genes, 38 tRNA genes, and a pseudogene. A phylogenetic tree was generated using the maximum-likelihood method, and the results revealed that *T. alba* was sister to *Pleione bulbocodioides* and *Pleione formosana*, with a support rate of 100%.

*Thunia alba* H. G. Reichenbach is a small genus belonging to family Orchidaceae, and it is composed of about six species. *Thunia* plants are distributed in Indonesia, Malaysia, Myanmar, Nepal, Thailand, Vietnam, India, Bhutan, and China, and *T. alba* is the only species found in China (Wu et al. 2009). *Thunia alba* is a beautiful orchid plant with showy white flower, and it can be cultivated as an epiphyte or terrestrial with humus-rich soil (Mathew 2013). In this study, the chloroplast genome of *T. alba* was assembled, and a phylogenetic was constructed to reveal its relationship with other species in the family Orchidaceae.

Fresh leaves were collected in an orchid nursery in Dongming (28°35′51″N, 121°18′48″E), Taizhou, Zhejiang, China. The CTAB method was applied to extract leaf DNA following the protocol (Doyle and Doyle 1987). A voucher specimen (PNT2018016) is stored at the Plant Physiology Laboratory in Jinhua Polytechnic. A DNA library was prepared and then sequenced using an Illumina Hiseq X Ten system (Illumina, San Diego, CA, USA). Totally 5.9 Gb clean reads were obtained by removing low-quality sequences with NGSQCToolkit version 2.3.3 (Patel and Jain 2012), and they were assembled using NOVOPlasty (Dierckxsens et al. 2017). The genome was annotated by Dual Organellar GenoMe Annotator (Wyman et al. 2004) with manual verification through BLAST searches, while the tRNA genes were predicted using the tRNAscan-SE version 2.0 program (Chan and Lowe 2019). The complete chloroplast genome (GenBank accession: MN527334) was 159,948 bp in size, containing a large single copy (LSC), a small single copy (SSC), and a pair of inverted repeats (IRs). The overall GC content of the whole genome was 37.2%. The lengths of LSC, SSC, and IR were 87,532, 18,852, and 26,781 bp, respectively, and their relative GC contents were 35.0%, 30.3%, and 43.2%.

The plastome was predicted to contain 135 genes, of which included 88 protein-coding genes, eight rDNA genes, 38 tRNA genes, and a pseudogene. Nine protein-coding genes (*ycf2*, *ycf15*, *ycf1*, *rps7*, *rps19*, *rps12*, *rpl23*, *rpl2*, and *ndhB*), four rRNA genes (*rrn5*, *rrn4.5*, *rrn23*, and *rrn16*), and seven tRNA genes (*trnV-GAC*, *trnR-ACG*, *trnL-CAA*, *trnI-GAU*, *trnI-CAU*, *trnH-GUG*, and *trnA-UGC*) were present in two copies. One copy of *ycf1* locating at the border of IR/SSC was identified as a pseudogene with a size of 1035 bp, while the normal *ycf1* was 5568 bp in length, encoding 1856 amino acids. Among the protein-coding genes, both *clpP* and *rps12* possessed two introns, and the other 11 genes, *rpl2*, *ndhB*, *ndhA*, *ndhB*, *rpl2*, *rpl16*, *petD*, *petB*, *rpoC1*, *atpF*, and *rps16*, each contained one intron. Moreover, six tRNA genes, *trnA-UGC*, *trnG-GCC*, *trnI-GAU*, *trnK-UUU*, *trnL-UAA*, and *trnV-UAC*, also contained a single intron.

A total of 13 chloroplast genome sequences of *Orchidaceae* species were downloaded from NCBI. A phylogenetic tree was generated using maximum-likelihood method by PhyML version 3.1 (Guindon et al. 2010), with *Burmannia disticha* (Burmanniaceae) as an outgroup. The results revealed that *T. alba* was sister to *Pleione bulbocodioides* and *P. formosana*, with a support value of 100% (Figure 1).

**Figure 1.**
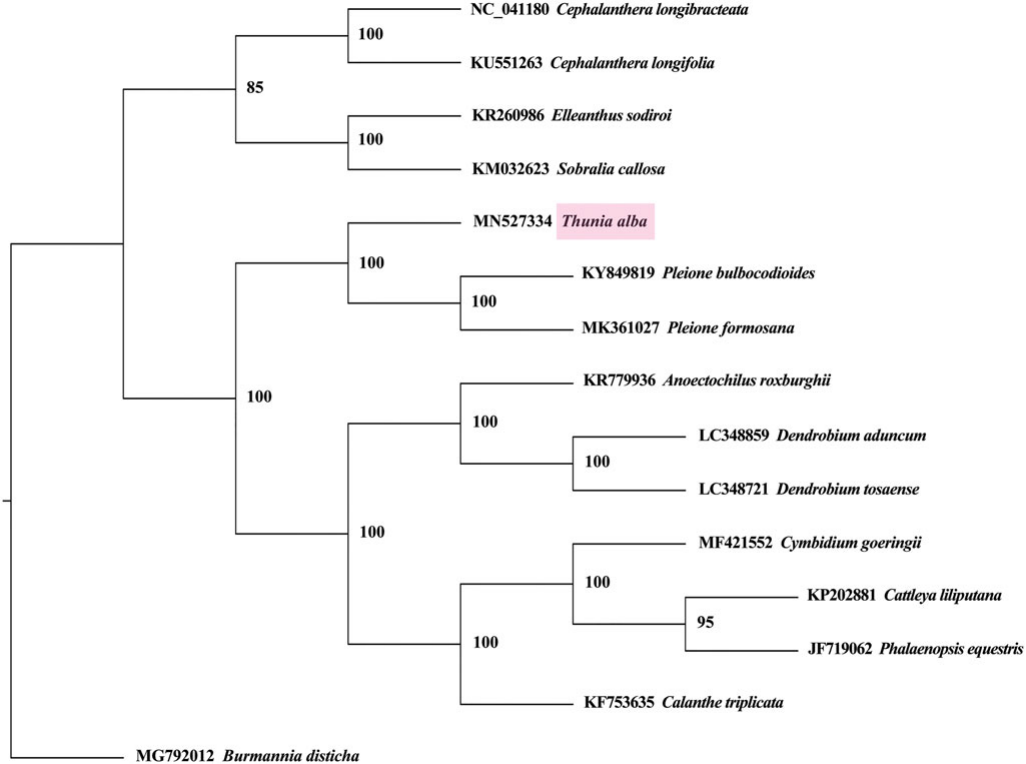
The maximum-likelihood tree based on complete chloroplast genome sequences of *Thunia alba* and 13 other Orchidaceae species, with *Burmannia disticha* (Burmanniaceae) as the outgroup. The numbers next to nodes indicate bootstrap support values.
